# Characterization of Vitellogenin and Vitellogenin Receptor of *Conopomorpha sinensis* Bradley and Their Responses to Sublethal Concentrations of Insecticide

**DOI:** 10.3389/fphys.2018.01250

**Published:** 2018-09-11

**Authors:** Qiong Yao, Shu Xu, Yizhi Dong, Yinli Que, Linfa Quan, Bingxu Chen

**Affiliations:** Plant Protection Research Institute, Guangdong Academy of Agricultural Sciences, Guangzhou, China

**Keywords:** *Conopomorpha sinensis* Bradley, vitellogenin, vitellogenin receptor, insecticides, sublethal effects

## Abstract

*Conopomorpha sinensis* Bradley is the dominant borer pest of *Litchi chinesis* and *Euphoria longan*. Current management of *C. sinensis* relies upon insecticide application to adult moths. In addition to the direct mortality induced by insecticides, a sublethal dose of insecticides also affects growth, survival, and reproduction in the exposed insects. Vitellogenin (Vg) and vitellogenin receptor (VgR) are normally identified as essential reproduction-related proteins in insects. In this study, we characterized these two genes from *C. sinensis*, and investigated their differential responses to sublethal concentrations of insecticide. Cloned CsVg and CsVgR consist of 5391 and 5424-bp open reading frames, which encode proteins of 1796 and 1807 amino acid residues, respectively. The CsVg protein contains the typical vitellogenin, DUF1943 and VWFD domains as other reported lepidopteran Vgs. The CsVgR was characterized as a typical low density lipoprotein receptor with two highly conserved LBD and EGF precursor domains, one hydrophobic transmembrane domain, one cytoplasmic domain, and 13 putative N-glycosylation sites. We next assessed the sublethal effect of four major insecticides on egg-laying in *C. sinensis*. The toxicity against *C. sinensis* varied among the insecticides tested, with LC_50_ values ranging from 0.23 ppm for chlorpyrifos to 20.00 ppm for β-cypermethrin, among which emamectin benzoate (EB) showed a significant negative impact on egg-laying, survival rate, ovarian development, and mating rate of *C. sinensis* at LC_30_ doses. Further investigation showed that the transcriptional level of CsVg and CsVgR were impaired in different way at 24, 48, and 72 h after EB exposure, and this result was in agreement with the diminished egg-laying of *C. sinensis* in the sublethal concentration EB-treated group. A repressed transcription level of CsVgR was observed at 48 h after treatment, suggesting that EB elicits a delayed response in the abundance of CsVgR. These results established different roles of CsVg and CsVgR in response to the sublethal effect of insecticides. CsVg might be a better parameter than CsVgR for assessing the effect of sublethal insecticides on reproduction in *C. sinensis*.

## Introduction

*Conopomorpha sinensis* Bradley (Lepidoptera: Gracilariidae) is the most destructive borer pest of *Litchi chinesis* and *Euphoria longan*, and causes severe economic loss in litchi and longan cultivated areas, including India, Nepal, Thailand, Vietnam, and China (Menzel, 2002; Schulte et al., 2007). Immediately after egg hatching, *C. sinensis* larvae bore tunnels into the center of tender shoots, flowers and fruits, and spend their larval stage inside host plants (Thanh et al., 2006; Dong et al., 2018). Thus, control of *C. sinensis* larvae is hampered due to its cryptic life habit and overlapping of generations in orchards. To date, frequent application of insecticides to adult *C. sinensis* is the most effective strategy for this borer pest, since the egg laying amount can be effectively reduced by decreasing the density of adult *C. sinensis* in orchards.

In addition to the direct mortality due to acute toxicity (lethal effect) after initial insecticide application, insects are exposed to low-lethal or sublethal doses of insecticides for a long period in fields (Biondi et al., 2013). Investigations of the acute toxicity and persistence of sublethal effects of insecticides are both needed for the assessment of insecticide efficiency. Sublethal doses of insecticides may affect various the physiological, biochemical, and behavioral traits of exposed insects (He et al., 2013; Abdu-Allah and Pittendrigh, 2018). The sublethal effects of insecticides on beneficial arthropods have received considerable attention, while their impacts on pest insects are poorly studied (Desneux et al., 2007; Liu et al., 2016). Actually, identifying the sublethal effects of insecticides on pest insects could help us better understand the overall insecticide efficacy in controlling the insect population, thereby optimizing the insecticide usage and in turn delaying resistance in pest insects.

Insecticide sublethal effects on reproduction of insects are traditionally been the most sublethal parameter studied for decades (Stark and Banks, 2003). LC25 of clothianidin could cause reduce mortality and reduction of egg-laying in *Bemisia tabaci* (Hemiptera: Aleyrodidae), but have no effect on oviposition duration and egg hatching rate of target pest insect (Fang et al., 2018). LC_30_ of buprofezin could significantly decrease the fecundity, longevity and egg hatchability in *Sogatella furcifera* (Hemiptera: Delphacidae) (Ali et al., 2017). While, LC_20_ of Cycloxaprid had no impact on net reproductive rage in *Aphis* *gossypii* (Hemiptera: Aphididae) (Cui et al., 2018). Thus, as a crucial parameter, the obtained reproduction related results are of primary importance for studies of insecticide sublethal effects on target pest insects. But limited literature had aimed to assessing insecticide sublethal effects on reproduction-related molecules of target insects. Vitellogenin (Vg) and vitellogenin receptor (VgR) are normally identified as two of the most important reproduction-related proteins in insects (Roth and Khalaila, 2012; Lee et al., 2017). Vg is the precursor reserve protein of the main yolk protein vitellin (Vn) in all oviparous species, including insects. The synthesis of Vg is extraovarian in origin with a stage-specific manner in the fat body of female insects. After releasing into the hemolymph and transportation to the ovaries, Vg is selectively accumulated by the terminal oocytes via a receptor-mediated endocytosis process. The receptor responsible for vitellogenin uptake is a membrane-bound protein (VgR) that is a member of the low density lipoprotein receptor (LDLR) family (Sappington and Raikhel, 1998; Roth and Khalaila, 2012). In addition to endocrine control, expression of Vgs and VgRs could be disturbed by several extraneous factors, including metal contamination stress, nutritional conditions, infection and chemical exposure. Previtellogenic nutrition alters the expression of VgR in *Aedes aegypti* (Hymenoptera: Apidae) (Clifton and Noriega, 2012). Both transcription and protein levels of Vg and VgR were down-regulated by bacterial infection in *Apis mellifera* (Hymenoptera: Apidae) (Abbo et al., 2017). Intriguingly, the expression of Vgs is dynamic and varies after exposure to different insecticides; e.g., Vg was decreased in a chlorpyrifos-resistant strain and identified as a novel potential resistance-related protein in *Frankliniella occidentalis* (Thysanoptera: Thripidae), while a drastic increase in the abundance of Vg was observed after exposure of sublethal concentrations of triazophos and deltamethrin in *Cyrtorhinus lividipennis* Ruter (Hemiptera: Miridae) (Yan et al., 2015; Lu et al., 2017). Hence, the identification and characterization of Vg and VgR, as well as the determination of their expression after exposure to sublethal doses of different insecticides, need to be established in each species to better understand the molecular mechanism of the sublethal effects of insecticides on reproduction in insects.

Here, we report the first molecular information on Vg and VgR in *C. sinensis*, the most destructive pest in the litchi and longan industry in China. In addition, the sublethal effects of different insecticides on egg-laying in *C. sinensis* were analyzed. Further, the transcript abundance of CsVg and CsVgR, as well as the survival rate, egg hatchability, ovary development, and mating rate of *C. sinensis* were investigated after treatment with different sublethal concentrations of emamectin benzoate (EB). This work aimed to provide richer molecular information for insect reproduction-related proteins, as well as to provide a more comprehensive picture of the relationship between sublethal effects of insecticides and reproduction in insects.

## Materials and Methods

### Insect Rearing and Collection

*Conopomorpha sinensis* pupae were collected as described earlier (Yao et al., 2016). One day-old female and male adult moths were raised separately for later use. The rearing condition were: constant-temperature incubation at 26 ± 1°C (temperature), 65–85% RH (relative humidity), 14:10 h L: D photoperiod and 20% (v: v) diluted honey.

### Identification of the Vitellogenin (CsVg) and Vitellogenin Receptor (CsVgR) Gene in *Conopomorpha sinensis*

Total RNA was extracted from five female adults of *C. sinensis* using the E.Z.N.A. Total RNA kit I (Omega Bio-tek, Norcross, GA, United States) and treated with DNase I (Omega). The RNA sample was dissolved in diethylpyrocarbonate (DEPC)-treated H_2_O and the RNA integrity was confirmed using agarose gel electrophoresis. First-strand cDNA was synthesized from 1 μg of total RNA in a 20 μl reaction mixture using the GoScript Reverse Transcriptase kit (Promega, Madison, WI, United States).

Four pairs of degenerate primers (CsVg-F1/CsVg-R1, CsVg-F2/CsVg-R2, CsVgR-F1/CsVgR-R1, and CsVgR-F2/CsVgR-R2) (**Supplementary Table 2**) were designed on the basis of the conserved Vg and VgR cDNA sequences of other Lepidoptera insects. PCR was performed to obtain partial cDNA sequences using TransTaq DNA Polymerase High Fidelity (Transgene Biotech, Beijing, China). PCR amplification was carried out as follows: 94°C for 5 min; five cycles of 94°C for 40 s, 48°C for 1 min and 72°C for 40 s; 25 cycles of 94°C for 40 s, 53°C for 1 min and 72°C for 40 s; with a final extension at 72°C for 6 min. The amplified products were separated on agarose gels and purified using a Gel Extraction kit (Axygen Biosciences, Union City, CA, United States). The purified PCR products were sub-cloned into the pGEM-T Easy Vector (Promega, Tokyo, Japan) and transformed into *Escherichia coli* DH5α-competent cells (Tiangen, Beijing, China). Positive clones were confirmed by PCR and automated sequencing [The Beijing Genomics Institute (BGI), China].

To obtain the full-length CsVg and CsVgR coding regions, nested gene-specific primers for CsVg and CsVgR (**Supplementary Table 2**) were designed based on the partial cDNA sequence obtained as described above. Following the instructions of the SMART^TM^ RACE (rapid amplification of cDNA ends) cDNA Amplification kit (Clontech, Mountain View, CA, United States), 5′-RACE and 3′-RACE were performed using gene-specific primers and universal anchor primers (Universal Primer Mix/UPM and Nested Universal Primer A/NUP, Clontech). The RACE products were confirmed using agarose gel electrophoresis and purified, sub-cloned into vectors and sequenced as described above. The overlapping sequences of the PCR fragments were assembled to obtain the full-length CsVg and CsVgR cDNA. Each kit was used according to the manufacturer’s instructions.

### Characterization of CsVg and CsVgR

The open reading frames of the CsVg and CsVgR genes were obtained using ORF finder^1^, and the amino acid sequences were deduced from the corresponding cDNA sequences using the translation tool on the ExPASy Proteomics website^2^. Various physical and chemical parameters for the CsVg and CsVgR proteins (such as predictions of the theoretical isoelectric point, molecular weight, theoretical isoionic point) were performed with analysis tools from the ExPASy ProtParam tool^3^. The signal peptide cleavage site was predicted using SignalP 4.1 Server^4^. The transmembrane helices were analyzed by TMHMM Server v.2.0^5^. Cellular localization was predicted by PSORT II^6^. The N-glycosylation site prediction was performed using the NetNglyc webserver^7^. The GPP prediction server was used to predict potential O-linked glycosylation sites^8^. The domain architecture and conserved domains were identified using Scan-Prosite^9^, SMART^10^ and InterProScan^11^ online tools. On the National Centre for Biotechnology Information (NCBI) website, the sequence of the CsVg and CsVgR cDNAs were individually compared with other available Lepidoptera vitellogenin and vitellogenin receptor sequences deposited in GenBank using the BLAST-X tool. Multiple sequence alignments of the deduced CsVg and CsVgR amino acid sequences were made using Multiple Alignment software^12^. Phylogenic and evolutionary analyses were conducted using Molecular Evolutionary Genetics Analysis (MEGA) software v.5.05 by a neighbor-joining (NJ) method with bootstrap of 1000 replicates after removing the highly divergent signal peptide sequences at the N-terminus.

### Sublethal Effect of Insecticides on Egg-Laying in *C. sinensis*

#### Sublethal Toxicity Test

A glass-vial bioassay was modified based on the scintillation glass-vial bioassay described in Snodgrass (Snodgrass, 1996) and was used in sublethal toxicity tests on adult *C. sinensis* in this study. This method allows for rapid dosing of large numbers of insects using small amounts of insecticides. Stock solutions of the four most common insecticides used in *C. sinensis* control, including chlorpyrifos, emamectin benzoate (EB), triazophos and β-cypermethrin (Sigma-Aldrich, St. Louis, MO, United States and Siminuo, Beijing, China), were obtained by dissolving these insecticides in acetone. Aliquots of these stock solutions were diluted with acetone to yield the desired concentrations for the bioassay. The concentration range of the test solutions varied for each insecticide, as follows: chlorpyrifos, 0.75–12.00 mg/kg; EB, 0.50–8.00 mg/kg; triazophos, 0.04–25.00 mg/kg, and β-cypermethrin, 0.13–18.00.

The insecticides were applied by pipetting 1 ml of acetone containing the test solutions into 500 ml beaker flasks, while the control breaker flask received only 1 ml of acetone. The beaker flask was rolled on its side until its interior surface was coated with an even layer of insecticide solution and dried at room temperature before treatment. Groups of 30 4-day-old adult *C. sinensis* (15 females and 15 males) were placed in each insecticide-treated and control beaker flasks. The bottleneck of the beaker flask was covered with medical gauze, and food for adult *C. sinensis* was provided by adding a cotton ball dipped in diluted honey. Beaker flasks were stored upright in the laboratory, and mortality was determined after 24 h. All bioassays were repeated three times. Mortality for the treated group was corrected for natural mortality in the control group using Abbott’s formula. All data were subjected to Probit analysis using PROC PROBIT (SAS Institute, 2008), generating a concentration-mortality regression line for each chemical.

#### Egg-Laying

Based on the regression equations obtained in this study, the LC_10_ and LC_30_ values of the four insecticides were used to evaluate their sublethal effects on egg-laying in *C. sinensis* (**Table 1**). Four-day-old *C. sinensis* females and males were selected randomly to be exposed to LC_10_ and LC_30_ values of the tested insecticides using the modified glass-vial bioassay described above. Ten pairs of surviving *C. sinensis* females and males were transferred from beaker flasks into insect rearing cages after 6 h. Each cage was considered to be a replicate and each treatment had three replicated cages. The replacement of oviposition stimulants (i.e., fresh litchi fruits), collection of eggs, and removal of dead *C. sinensis* were carried out every 24 h for 3 days.

**Table 1:** LC_50_ values (with corresponding 95% confidence intervals) for *Conopomorpha sinensis* adults after 24 h of exposure to insecticides.

Insecticides	Regression equations	*X*^2^(df)	LC_50_ (ppm)	CI	LC_30_ (95%CL)	LC_10_ (95%CL)
Chlorpyrifos	y = 6.41+2.24x	7.96 (3)	0.23	0.18–0.30	0.14 (0.05–0.15)	0.06 (0.01–0.06)
Emamectin benzoate	y = 4.68+1.17x	0.24 (3)	1.88	0.99–3.41	0.67 (0.16–1.21)	0.15 (0.01–0.41)
Triazophos	y = 4.11+2.74x	0.12 (3)	2.11	1.43–4.03	1.36 (0.78–1.95)	0.72 (0.30–1.13)
β-Cypermethrin	y = 3.71+0.99x	1.23 (3)	20.00	11.47–27.17	5.92 (1.97–10.28)	1.02 (0.04–2.66)

*The results are presented as regression equations, degree of freedom (df), LC_50_, corresponding 95% confidence intervals (CI), estimated LC_30_ and LC_10_. Low X^2^ value (<11.00) indicates the data adequacy to the probit model used to estimate the mortality curves.*

### Fecundity Analysis After Sublethal EB Exposure in *C. sinensis*

#### Survival Rate

Based on the effect of the tested insecticides on fecundity of *C. sinensis*, EB was selected to investigate the sublethal effect of the insecticide on the survival rate of *C. sinensis*. Briefly, Sixty 4-day-old *C. sinensis* adults were exposed to LC_10_ and LC_30_ doses of EB. Dead *C. sinensis* male and females were removed and counted every 24 h for 7 days.

#### Egg Hatchability

Eggs from each treatment were collected and kept separately for hatching assessment. Hatched larvae were counted daily until no larvae hatched for at least 48 h.

#### Ovary Dissection

To verify the influence of sublethal concentrations of EB on the ovary development of *C. sinensis*, ovaries of females were dissected in phosphate buffered saline (PBS) at 72 h after sublethal EB exposure. Dissected ovaries were washed and photographed.

#### Mating Rate

Ten females were dissected at 72 h after sublethal EB exposure. Mating rate was calculated based on the form of bursa copulatrix. Small and wizened bursa copulatrix indicates unmated female, while big and plump bursa copulatrix indicates mated female. Each treatment had three replicates.

#### Gene Expression

Quantitative real-time PCR (qRT-PCR) analysis was performed to determine the expression of CsVg and CsVgR in *C. sinensis* after exposure to sublethal doses of EB. Four-day-old *C. sinensis* adults were exposed to LC_10_ and LC_30_ doses of EB using the modified glass-vial bioassay described above. Sixty surviving *C. sinensis* were transferred from beaker flasks into insect rearing cages after 6 h. Five females were selected randomly from the treatment and control groups every 24 h for 3 days. All bioassays were repeated three times. Total RNA was isolated from total insects of individual samples (5 females/sample) and treated with DNase I (Omega). cDNA was synthesized from 1 μg of total RNA in a 20 μl reaction mixture using the GoScript Reverse Transcriptase kit (Promega, Madison, WI, United States) according to the manufacturer’s protocol. The mRNA transcripts of CsVg and CsVgR were assessed using the GoTaq qPCR Master Mix (Promega, Madison, WI, United States) with the specific primers described in **Supplementary Table 2**. PCR was performed using the specific primers mentioned in **Supplementary Table 2**. The PCR conditions were hot-start activation at 95°C for 2 min; 40 cycles of denaturation at 95°C for 15 s and extension at 60°C for 1 min; followed by a final dissociation at 72°C. The specificity of the reaction was checked by analyzing the melting curve of the final amplified product. *β-actin* (KF598848) was chosen as a suitable housekeeping gene, and the housekeeping gene and target genes from each sample were run in triplicate on the same PCR plate. The relative expression levels were calculated using a modified comparative Ct method (Schmittgen and Livak, 2008), and the relative expression levels of the CsVg and CsVgR genes were calculated by normalization to β-actin.

### Statistical Analysis

The LC_50_ ratio for each insecticide was tested for significance according to Robertson and Preisler (1992) to determine differences at *P* > 0.05, which was achieved by calculating the corresponding 95% confidence intervals (Robertson and Preisler, 1992). The daily fecundity for each egg collection time was analyzed separately using one-way analysis of variance (ANOVA) with the type of insecticide as the independent variable. Then, multiple comparison procedures were performed by Tukey’s test when significant differences were found (*P* < 0.05). For all experimental data, statistical analyses were performed by ANOVA followed by Tukey’s test for multiple comparisons. Significant differences were considered at *P* < 0.05.

## Results

### Sequence and Structural Analysis of CsVg

Cloning of CsVg was accomplished by RT-PCR using degenerate primers (CsVg-F1, CsVg-R1, CsVg-F2, and CsVg-R2; sequences in **Supplementary Table 2**) designed based on the conserved amino acid regions of other lepidopteran Vg genes. Two cDNA fragments of 834 and 1075 bp were identified, and one full-length cDNA was obtained with a combination of 3′ and 5′ RACE technologies using two pairs of nested gene-specific primers based on the cDNA fragments mentioned above. The deduced protein of CsVg is composed of 1796 amino acids and had a signal peptide (MKVLVLAALLAAASC) at the N-terminus. The deduced CsVg protein is predicted to be an unstable protein with a calculated molecular mass of 205.8 kDa and a pI value of 7.99 (**Supplementary Table 1**).

**FIGURE 1:**
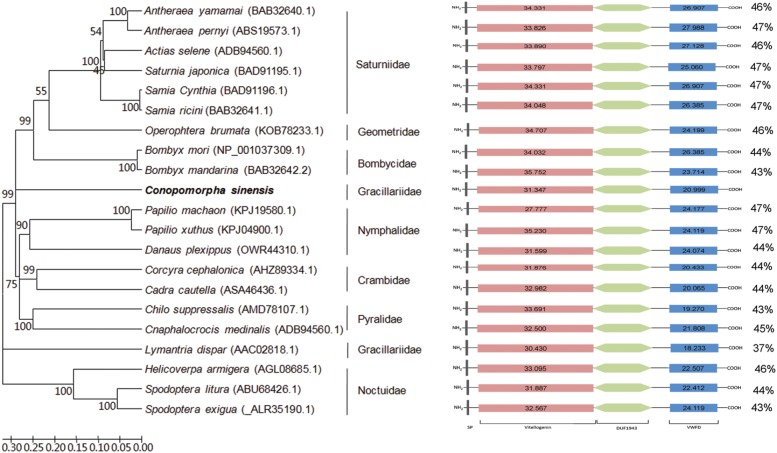
Domain architecture and phylogenetic analysis of lepidopteran vitellogenins (Vgs). A phylogenetic tree was constructed using the sequences of different lepidoptera families. The *Conopomorpha sinensis* sequence was used as an outlier, and the figure shows a family grouping of the lepidopteran Vgs. The figure shows a comparable domain architecture in lepidopteran Vgs. The percentages on the right indicate the overall identity of each protein compared to CsVg. Scan-Prosite scores for vitellogenin and VWFD domains of Vgs are presented in the schema. DUF1943, Vitellinogen, open beta-sheet; VWFD, von Willebrand factor, type D domain; SP, signal peptide.

Three domains and several exposed functional residues were identified in CsVg, which are highly conserved in the sequenced Vgs of Lepidoptera. Domain architecture analysis by the Scan-prosite and InterProScan server confidently predicted the presence of three functional domains in CsVg (**Figure 1**). The Vitellogenin domain (PS51211), also called the lipoprotein amino-terminal region or LPD_N, is shown to span amino acids 40–752. The DUF1943 domain, which was rarely studied and of unknown function, is in the middle of CsVg (spanning amino acids 784–1062). The Von Willebrand Factor type D domain (VWFD domain, PS51233) is positioned near the C-terminus of the CsVg, from amino acids (aa) 1447 to 1627. Several tetra residue motifs, R/KXXR/K, are present in CsVg at the N-terminus and in the middle of CsVg (**Supplementary File 1**). In addition, a highly conserved GL/ICG motif and five cysteine residues are located at conserved positions, as in other reported insect Vgs.

The deduced CsVg was aligned with corresponding amino acid sequences of other lepidopteran Vgs by NCBI protein BLAST. The results revealed that CsVg had low a degree of conservation with other lepidopteran Vgs, with the overall identity ranked from 47 to 37%. The evolutionary relationship of 21 Vgs derived from lepidopteran insects was evaluated after sequence alignment and phylogenetic tree construction. The monophyly of nine families was well-supported by an NJ tree with high values, with CsVg belonging to Gracillariidae located in a separated branch.

### Sequence and Structural Analysis of CsVgR

Several vitellogenin receptors (VgRs) were identified from different families of lepidopteran insects, such as *Bombyxi mori* (Lepidoptera: bombycidae), *Spodoptera exigua* (Lepidoptera: noctuidae), and others. However, the VgR protein from gracilariidae was not previously characterized. Therefore, we cloned the gene encoding the VgR protein from *C. sinensis* using a similar strategy as that used for CsVg cloning. Two CsVgR fragments of 1221 and 525 bp were generated from adult *C. sinensis* cDNA by RT-PCR using two pairs of degenerate primers (CsVgR-F1, CsVgR-R1, CsVgR-F2, and CsVgR-R2), and then full-length cDNA of CsVgR was obtained using two pairs of specific primers (sequences in **Supplementary Table 2**). The deduced protein of CsVgR is composed of 1807 amino acids with a predicted molecular mass of 201.2 kDa, a pI value of 5.57, and the highest aa composition of serine (**Supplementary Table 1** and **Supplementary File 2**). Analysis of the deduced amino acid sequence revealed a signal peptide with 20 aa residues (MSNKWLVTMITVSLCGVAWA) located at the N-terminus of CsVgR, which was presumed to be cytoplasmic in nature, as detected by SignalP 4.1 and PSORT II Server.

**FIGURE 2:**
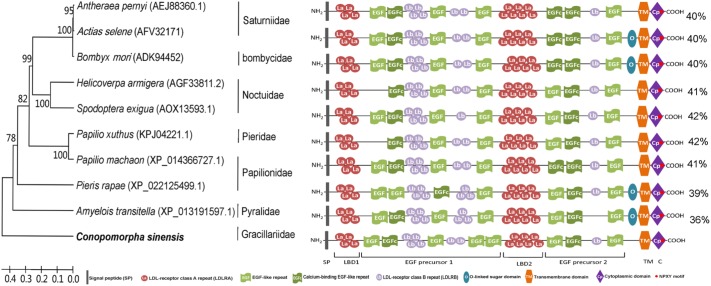
Domain architecture and phylogenetic analysis of lepidopteran vitellogenin receptors (VgRs). The phylogenetic tree was constructed using sequences of different lepidoptera families. The *C. sinensis* sequence was used as an outlier, and the figure shows family grouping of the lepidopteran VgRs. The figure shows a comparable domain architecture in insect VgRs from different lepidoptera families. The percentages on the right indicate the overall identity of each protein compared to CsVgR.

Analysis of the CsVgR protein sequence indicated that it contained all of the typical features of the LDLR family. The structural organization of mature insect VgRs consists of (i) two ligand-binding domains with LDL-receptor class A (LDLRA) repeats, (ii) two epidermal growth factor (EGF) precursor domains with EGF-like repeats and LDL-receptor class B (LDLRB) repeats, (iii) an O-linked sugar domain, (iv) a transmembrane domain, and (v) a cytoplasmic domain (Tufail and Takeda, 2009). In the architecture analysis of the mature CsVgR protein, we observed 11 cysteine-rich LDLRA repeats in two patches, 7 cysteine-rich LDLRB repeats in three patches, 6 EGF-like repeats, 2 calcium binding EGF-like repeats, one hydrophobic transmembrane domain, one cytoplasmic domain with two sequence motifs (NPLF at residues 1748–1751 and LL at residues 1756–1757) as potential receptor internalization signals, and 13 putative *N*-glycosylation sites characterized by the consensus sequence NXS/T (**Figure 2** and **Supplementary Table 3**). The deduced amino acid sequence of CsVgR was aligned with other lepidopteran VgRs by a blastp search of the NCBI database. The CsVgR protein sequence was most similar to those of VgR from *S. exigua* and *Papilio xuthus* (Lepidoptera: Papiloinidae) (42% overall identity), followed by *Helicoverpa armigera* (Lepidoptera: Noctuidae) and *Papilio machaon* (Lepidoptera: Papiloinidae) (41%) (**Figure 2**).

### Sublethal Effect of Insecticides on Egg-Laying in *C. sinensis*

**FIGURE 3:**
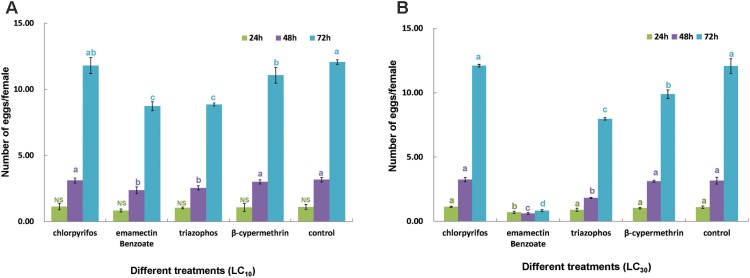
Sublethal effect of insecticides on fecundity in *C. sinensis.* Four insecticides (chlorpyrifos, emamectin benzoate, triazophos, and β-cypermethrin) were used to evaluate their sublethal effects on egg-laying in *C. sinensis* at the concentrations of LC_10_ **(A)** and LC_30_ **(B)**. Daily fecundity was analyzed separately for 3 days. Data for each egg collection time were analyzed separately using one-way analysis of variance (ANOVA) with type of the insecticides as the independent variable. Then, multiple comparison procedures were performed via Tukey’s test. Different lowercase letters in different colors above the same color columns indicate significant differences at each egg collection time (24, 48, and 72 h) (*P* < 0.05).

Probit analyses of concentration-mortality data showed that after 24 h of exposure to chlorpyrifos, EB, triazophos and β-cypermethrin, the LC_50_ values were estimated to be 0.23, 1.88, 2.11, and 20.00 ppm, respectively (**Table 1**). These results revealed that chlorpyrifos had the highest toxicity to *C. sinensis*, followed by EB and triazophos, whose LC_50_ values were more than 10 times higher than that of β-cypermethrin. The LC_10_ and LC_30_ values of each insecticide to adult *C. sinensis* were estimated based on the regression equations of the four insecticides (**Table 1**). The egg-laying of *C. sinensis* females after exposure to sublethal insecticides was determined. *C. sinensis* females in the EB-treated group laid the fewest eggs among the four insecticide-treated groups, followed by the triazophos-treated group. The other two insecticides showed little to no effect on egg-laying in *C. sinensis* (**Figure 3**). Moreover, the average number of eggs laid per female in control group was five times and 14 times higher than that of the LC_30_ EB-treated group at 48 and 72 h after chemical exposure, respectively. Therefore, taking the results of toxicity and impact on oviposition in *C. sinensis* into account, the most detrimental chemical is with EB. Thus, EB was selected to further investigate the sublethal effect of insecticide on the fecundity of *C. sinensis*.

### Sublethal Effects of EB on Fecundity in *C. sinensis*

**FIGURE 4:**
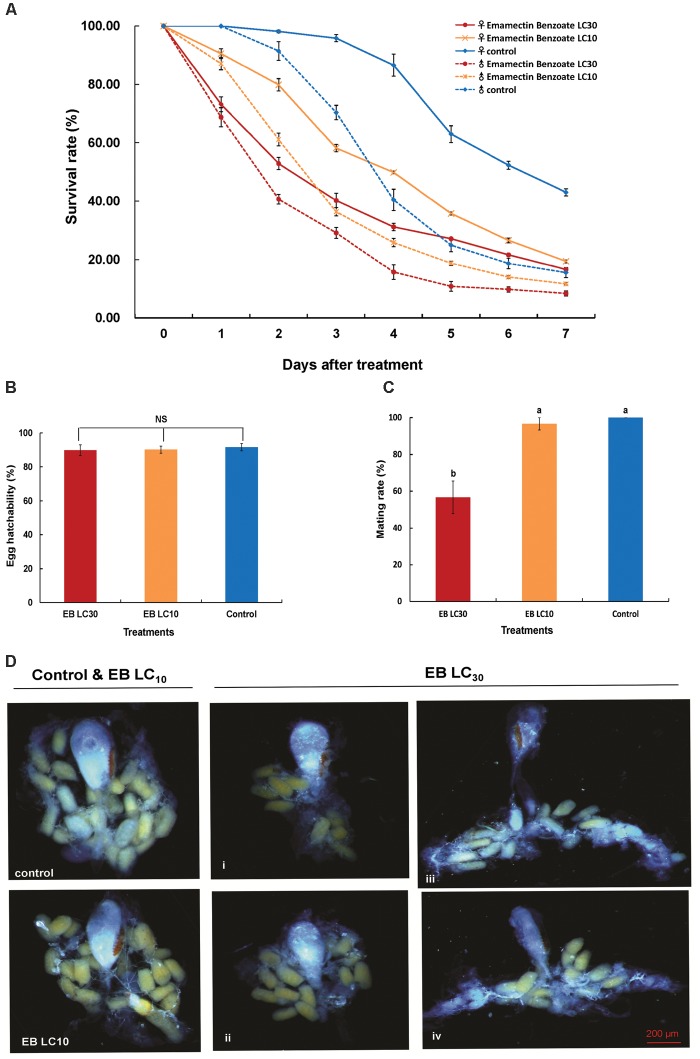
Effects of emamectin benzoate (EB) exposure on the survival rate, egg hatchability, mating rate, and ovarian development in *C. sinensis* at the sublethal concentrations. The figure shows the survival rate **(A)**, egg hatchability **(B)**, mating rate **(C)**, and ovarian development **(D)** in *C. sinensis* at different treatments (LC_10_ EB, LC_30_ EB, and Control). The bars represent the average ( ± SD). Different lowercase letters above the columns indicate significant differences (Tukey’s test, *P* < 0.05).

The two sublethal concentrations (LC_10_ and LC_30_) of EB caused diminished survival rates of adult *C. sinensis* males and females that were significantly different from the survival rates recorded for the control group (**Figure 4A**). The survival rates of adult *C. sinensis* at both LC_10_ and LC_30_ of the treatments exhibited a similar trend and had a sharp decline 2–3 days after EB exposure. Only 21.87% of females and 9.82% of male at LC_30_ concentration, and 23.79% of female and 19.12% of males at LC_10_ concentration were able to survive for 3 days post-chemical exposure. The survival rates of adult *C. sinensis* females were higher than those of males, indicating that female moths were more tolerant to EB. The mating rate was remarkably decreased relative to the control and L_10_ EB treatment group when *C. sinensis* adults were exposed to LC_30_ EB (**Figure 4C**). By contrast, egg hatchability was unaffected by EB exposure (**Figure 4B**). In the morphological photographs of ovaries of the *C. sinensis* females at 72 h after EB treatment, no significant difference was observed between the LC_10_ EB treatment and control groups, while ovaries with fewer eggs or undeveloped ovaries with small and wizened bursa copulatrix were observed after the LC_30_ EB exposure (**Figure 4D**).

### Sublethal Effects of EB on Transcription Level of CsVg and CsVgR in *C. sinensis*

**FIGURE 5:**
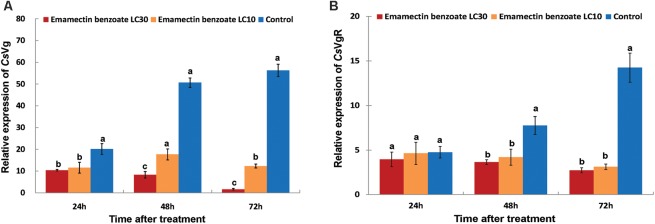
Effects of emamectin benzoate exposure (EB) on the gene expression of CsVg and CsVgR in *C. sinensis* at the sublethal concentrations. The figure shows the gene expression of CsVg **(A)** and CsVgR **(B)** in *C. sinensis* at different treatments (LC_10_ EB, LC_30_ EB, and Control). The bars represent the average ( ± SD) of the relative CsVg and CsVgR expression with *β-actin* as the housekeeping gene. Different lowercase letters above the columns indicate significant differences (Tukey’s test, *P* < 0.05).

To address the impact of sublethal concentrations of EB on gene expression of reproduction-related proteins in *C. sinensis*, the relative mRNA expressions of of CsVg and CsVgR were determined. Exposure of LC_10_ and LC_30_ EB to adult *C. sinensis* resulted in a significantly diminished transcriptional abundance of CsVg and CsVgR. The CsVg mRNA levels in insects from the LC_30_ EB-treated group stayed very low at 48 and 72 h after treatment, less than 30% of that in insects from the LC_10_ EB-treated group (**Figure 5A**). By contrast, no significant differences in the transcriptional abundances of CsVgR were observed at 48 and 72 h after the LC_10_ and LC_30_ EB treatment, indicating that EB had a lower adverse impact on CsVgR expression than CsVg in *C. sinensis* (**Figure 5**). It is interesting to note that a significantly decreased transcription level of CsVg was observed at 24 h after treatment, while a repressed transcription level of CsVgR was observed at 48 h after treatment.

## Discussion

Vgs and VgRs have been extensively identified in different species of vertebrates and invertebrates, including insects. To date, 20 Vg and 9 VgR sequences from lepidopteran are available in the GenBank database, but no information has been reported for Vg and its receptor from gracillariiae insects. In this study, we identified the sequences of Vg and VgR in *C. sinensis* (lepidoptera: gracillariiae), which are the first full-length sequences of Vg and VgR from the gracillariidae insects, and provided basic information for their functional analysis. The evolutionary relationship of CsVg and CsVgR with other lepidoteran insect Vgs and VgRs was inferred by constructing two phylogenetic trees. CsVg and CsVgR were separate from other lepidoteran insects and formed a single clade of gracillariidae, as expected.

Similar to other reported insect Vgs, CsVg led to a 205.8 kDa precursor protein with three functional domains, including highly a conserved vitellogenin domain and VWFD domain (Upadhyay et al., 2016). The DUF1943 domain, which is rarely detected in insects and is of unknown function, is present in all lepidopteran Vgs characterized to date (Robertson and Preisler, 1992; Thompson and Banaszak, 2002) (**Figure 1**). In the meantime, the CsVg protein sequence also contained different features from other insects. (1) The consensus cleavage sites of the R/KXXR/K tetra-residue motif are near the N-terminus of most insect Vg proteins (Tufail and Takeda, 2008). However, multiple potential R/KXXR/K sequence motifs are found at the N-terminus, center and C-terminus of Vg in *C. sinensis*. (2) Most insect Vgs characterized to date are heavily phosphorylated, especially at serine regions. Polyserine tracts containing tandem serine repeats are mostly present at both of the termini in insect Vgs (Tufail and Takeda, 2008). These polyserine tracts are expected to serve as good phosphorylation sites and may contribute to the interaction between Vg and VgR during endocytosis (Goulas et al., 1996; Upadhyay et al., 2016). Although 315 putative phosphorylated residues (S = 149, Y = 103, and T = 99) were predicted in CsVg, polyserine tracts were not observed in CsVg as well as missing polyserine tracts reported in *S. exigua*, indicating that there may be different mechanism for Vg and VgR binding on the oocyte surface in some insects, such as *C. sinensis* and *S. exigua* (Zhao et al., 2016). (3) In most of the insect Vg sequences, DGXR motif, conserved cysteine residues, and GI/LCG motif occur at highly conserved locations near the C-terminus, and the DGXR motif is normally located 17–19 residues upstream of the GI/LCG motif (Sun et al., 2016). It is proposed that they may form a structure that is necessary for the proper function of insect Vgs during embryogenesis. However, no DGXR motif was detected upstream of the GLCG motif in CsVg, as reported previously for *L. maderae* Vg, indicating the alternation of the DGXR motif in some insect species (Tufail and Takeda, 2008).

Analysis of the CsVgR sequence showed that it was composed of multiple conserved modular elements, similar to other insect VgRs and was a typical member of the LDLR superfamily (Sappington and Raikhel, 1998). A striking characteristic of lepidopteran VgRs is the existence of 11 cysteine-rich LDLRA repeats in two LBD domains, which are four and seven repeats in the first and second LBD domain, respectively. However, the number of LDLRA repeats and arrangement are quite different from those other insect orders, There are five- and eight- LDLRA repeats in Blattaria and Diptera, two/four- and eight- repeats in Hymenoptera, and eight-repeats in Coleoptera (Cong et al., 2015; Zhang et al., 2016). Moreover, we found comparable patterns in each lepidoteran VgR, although the numbers of conserved modular elements were variable. Lepidoteran VgRs all contain two ligand binding domain (LBD) domains and two EGF precursor domains that are responsible for ligand binding and acid-dependent dissociation; each LBD domain is followed by an EGF precursor domain (Tufail and Takeda, 2009). However, compared with other lepidopteran VgRs, the number and arrangement of EGF/Calcium-binding-like repeats in the two EGF precursor domains are different in *C. sinensis*. In common, two/three EGF-like repeats were presented in the first EGF precursor domain, but an extra repeat was observed in the *C. sinensis* VgR. In addition, the numbers of EGF/Calcium-binding EGF-like repeats in the second EGF precursor domain in lepidoteran VgRs varied substantially (**Figure 2**). Another intriguing difference among lepidopteran VgRs is the existence of the O-linked sugar domain, which is a short serine and threonine enriched region at the C-terminus of some insect LDLRs. It is proposed that the O-linked sugar domain is important for VgR stability and regulation of the signal pathway (Willnow, 1999; Tufail and Takeda, 2009). However, the CsVgR do not contain an O-linked sugar domain, as in *Antheraea pernyi* (Lepidoptera: Saturniidae), which is different from the VgRs of *Actias selene* (Lepidoptera: Saturniidae) and *Bombyx mori* (Lepidoptera: Saturniidae) (**Figure 2**). These results indicate that the presence of the O-linked sugar domain is not universal even among the same insect family.

Emamectin benzoate (EB) is a macrocyclic lactone insecticide and acts by disrupting the nervous system, inhibiting muscle contraction, damaging the detoxic ability, and thereby leading to changes in metabolism and behavior of pest insects (Isaac et al., 2002; Luan et al., 2017). With long residual ingestion activity on target arthropods and low toxicity to beneficial arthropods, EB is widely used for control of pest insects (López et al., 2010). In previous research, EB exhibited ovicidal activity against *Cydia molesta* (Busck) (Lepidoptera: Tortricideae), larvicidal activity against *Culex quinquefasciatus* say (Diptera: Culicidae), and adulticidal activity against *Cydia pomonella* (L.) (lepidoptera: Tortricidae) (Ioriatti et al., 2009; Wu et al., 2015; Shah et al., 2016). However, EB was reported to be a harmless insecticide for adults of *Adalia bipunctata* (L.) (Coleoptera: Coccinellidae), *Coccinella transversalis* (F.) (Coleoptera: Coccinellidae), and *Macrolophus pygmaeus* (Hemiptera: Miridae) (Cole et al., 2010; Martinou et al., 2014; Depalo et al., 2017). Thus, the effectiveness of EB against insects is species- and phase- dependent. In the current study, the sublethal concentration of EB on the survival rate of adult *C. sinensis* was evaluated under laboratory conditions. The LC_30_ concentration of EB had a long-lasting toxic activity and reduced the survival rate by ∼50% in adult *C. sinensis* 2 to 4 days after treatment. These results are consistent with the field application of EB in litchi and longan orchards; i.e., high mortality of *C. sinensis* moths is observed 3–4 days after EB spraying, and the application of EB decreases the pest population and achieves a long-term pest population decrease (Shu Xu, unpublished). These results demonstrated that EB was a long-lived insecticide for the adult *C. sinensis* control. Insecticides, as an environmental hazard, can affect insect reproduction by directly and indirectly linking with insect population via physiological and biochemical pathways (He et al., 2013; Roditakis et al., 2013). Among the four tested insecticides, the sublethal concentrations of EB showed a significant negative impact on egg-laying in *C. sinensis* at 48 and 72 h after chemical exposure. Moreover, ovarian development was disturbed and mating rate of *C. sinensis* was decreased to 56.67% after LC_30_ EB exposure. Likewise, a notable reduction in the mating frequency in female *Helicoverpa zea* (Lepidoptera: Noctiudae) was observed after treatment with sublethal concentrations of EB (López et al., 2010). Therefore, the influence of sublethal concentrations of EB on insect mating behavior could be a possible explanation available for the impact of this hazardous chemical on oviposition in *C. sinensis*.

Among all insect reproduction-related proteins, Vg and VgR have traditionally been used as adequate parameters for assessing female fertility (Lee et al., 2017; Seixas et al., 2018). In our study, the transcriptional abundance of CsVg and CsVgR in insects from the control group was increased at 6 and 7 days after eclosion of *C. sinensis* (sampling time of 48 and 72 h in the control group, **Figure 5**). These results are in agreement with the readiness of the female *C. sinensis* for mating and oviposition after preoviposition period for 5 days in our previous study (Dong et al., 2015). On the contrary, the transcript levels of CsVg and CsVgR were generally decreased in different ways at 48 and 72 h after EB exposure, and this result was coincident with the diminished egg-laying of *C. sinensis* in the LC_10_ and LC_30_ EB-treated groups. Interestingly, EB down regulated the expression CsVg, but left the transcriptional level of CsVgR undisturbed in the initial 24 h after EB exposure. In previous studies, exposure of 3^rd^ instar larvae to sublethal concentrations of chlorantraniliprole resulted in decreased fecundity and Vg expression in adult *Chilo suppressalis* (Lepidoptera: Crambidae) females; exposure to sublethal doses of both fipronil and deltamethrin did not affect Vg expression in *Apis mellifera* (Hymenoptera: Apidae) while induced Vg expression was observed in *Nilaparvata lugens* (Hemiptera: Delphacidae) after a sublethal deltamethrin treatment (Ge et al., 2010; Li et al., 2016; Bordier et al., 2017). Therefore the expression of Vg in different species of insects varied substantially after sublethal insecticide exposure. Our findings provide evidence that EB elicited an important response in adult *C. sinensis* females by modulating the expression of CsVg and CsVgR.

## Conclusion

This study molecularly characterized CsVg and CsVgR in *C. sinensis*, and is the first report of Vg and its receptor in gracillariiaes insects. In addition, the primary toxicity and fecundity regulation of insecticides on *C. sinensis* and expression response of CsVg and CsVgR to EB exposure were also investigated. Thereby further our understanding of the role of the Vg and VgR genes in insecticide-responsive gene expression. Its more timely response and more drastic reduction in the abundance of CsVg than CsVgR indicated that CsVg might be a better parameter for the assessment of sublethal insecticide impacts on reproduction in target insects. Our results demonstrated that EB could be considered an effective insecticide with high persistence for controlling *C. sinensis*. However, more detailed studies of non-target beneficial arthropods, and adaptation and resistance to long-term EB exposure need to be carried out for a comprehensive understanding of the influence of EB application in litchi and longan orchards.

## Author Contributions

QY, SX, and BC conceived the study. QY conducted the experiments and drafted the preliminary manuscript. QY, YQ, YD, and LQ interpreted the results. SX and BC refined and approved the final manuscript.

## Conflict of Interest Statement

The authors declare that the research was conducted in the absence of any commercial or financial relationships that could be construed as a potential conflict of interest.

## Footnotes

1 https://www.ncbi.nlm.nih.gov/orffinder/

2 http://expasy.org/tools/dna.html

3 https://web.expasy.org/protparam/

4 http://www.cbs.dtu.dk/services/SignalP/

5 http://www.cbs.dtu.dk/services/TMHMM/

6 http://psort.hgc.jp/form2.html

7 http://www.cbs.dtu.dk/services

8 http://comp.chem.nottingham.ac.uk/glyco/

9 http://prosite.expasy.org/scanprosite/

10 http://smart.embl-heidelberg.de/

11 http://www.ebi.ac.uk/Tools/pfa/iprscan/

12 http://www.ebi.ac.uk/clustalw/index.html
