# Supplementary material for: Characterization of Vitellogenin and Vitellogenin Receptor of Conopomorpha sinensis Bradley and Their Responses to Sublethal Concentrations of Insecticide
Source: Front Physiol. 2018 Sep 11;9:1250. doi: 10.3389/fphys.2018.01250 (PMC6154279; doi:10.3389/fphys.2018.01250)
Supplement: Supplementary file 1 [file Table_1.DOCX]

**Supplementary file 1 table**. Details of *C. sinensis* vitellogenin and vitellogenin receptor sequences

| Gene | Accession number | ORF (kb) | Protein (AAs) | MW  (kDa) | pI | Cellular localization | Highest AA composition | GRAVY | instability | SP (AAs) |
| --- | --- | --- | --- | --- | --- | --- | --- | --- | --- | --- |
| Vitellogenin | MH553377 | 5391 | 1796 | 205.8 | 7.99 | nuclear | Ser (S) 8.3% | -0.654 | unstable | 15 |
| Vitellogenin receptor | KX987145 | 5424 | 1807 | 201.2 | 5.57 | cytoplasmic | Ser (S) 8.0% | -0.384 | stable | 20 |

AA: Amino acid

MW: Molecular weight

GRAVY : Grand average of hydropathicity

pI : isoionic point

SP: signal peptiede
