# Supplementary material for: Characterization of Vitellogenin and Vitellogenin Receptor of Conopomorpha sinensis Bradley and Their Responses to Sublethal Concentrations of Insecticide
Source: Front Physiol. 2018 Sep 11;9:1250. doi: 10.3389/fphys.2018.01250 (PMC6154279; doi:10.3389/fphys.2018.01250)
Supplement: Supplementary file 2 [file Table_2.DOCX]

**Supplementary file 2 table.** primers used in this study.

| For Vg or VgR of *Conopomorpha sinensis* cloning | | |
| --- | --- | --- |
| Name of primers | Degenerate primers (5’-3’) | |
| CsVg-F1 | TGGARACHGAYRTNACHGGYGA | |
| CsVg-R1 | GGCATDTTRTTRGGRTTYTG | |
| CsVg-F2 | CMYMTGADGTNMGRGTKGCNGC | |
| CsVg-R2 | GCASWRTANGGCCANACRC | |
| CsVgR-F1 | TCGAYATGGAYTAYGAYTACG | |
| CsVgR-R1 | ARTMKATAMBRCCGTTKCYTTC | |
| CsVgR-F2 | TGYGHSGACATBRACGAGTG | |
| CsVgR-R2 | TCNRCRGCYARHCCSGWGC | |
| Name of primers | Nested gene-specific primers (5’-3’) | |
| 5- CsVg-R3 | ATCGAATGCATCATAGCTG | |
| 5- CsVg-R4 | TGAGTGAGCTGTTCCAGTCC | |
| 5- CsVgR-R3 | AGCTCATCCTGCTGGGTTAC | |
| 5- CsVgR-R4 | TGACAGGAGTACATAACGGC | |
| 3- CsVg-F3 | AGCATGAAACTGGTATCTACGG | |
| 3- CsVg-F4 | AAGCTACGCGTTCTACCTTC | |
| 3- CsVgR-F3 | TGCTCTCTCTGTTATGCCC | |
| 3- CsVgR-F4 | ACAACAATGGCAGTGGACCC | |
| For quantitative real-time RT-PCR | | |
| Gene | Forward (5’-3’) | Reverse (5’-3’) |
| Vitellogenin | ATGCAACCATAGAGTCGC | ATCATCTTCCGTAGATACCAG |
| Vitellogenin receptor | ACACTACATCCGCAGGCTG | TGACAGGAGTACATAACGGCC |
| 𝛽-Actin | AGATCTGGCACCACACCT | ACGATACCGGTGGTACGAC |
