# Supplementary material for: Characterization of Vitellogenin and Vitellogenin Receptor of Conopomorpha sinensis Bradley and Their Responses to Sublethal Concentrations of Insecticide
Source: Front Physiol. 2018 Sep 11;9:1250. doi: 10.3389/fphys.2018.01250 (PMC6154279; doi:10.3389/fphys.2018.01250)
Supplement: Supplementary file 3 [file Table_3.DOCX]

**Supplementary file 3 table**. Confidently predicted domains or motifs of *C. sinensis* vitellogenin receptor (*Cs*VgR) protein by SMART server

| Domains or motifs | Start-End |
| --- | --- |
| Signal peptide | 1-20 |
| LDLa | 32-70,77-116,125-163,177-222 |
| EGF | 226-263 |
| EGF_CA | 264-288 |
| LY | 330-372,375-417,418-460,461-500 |
| EGF | 571-605 |
| LY | 682-725 |
| EGF | 892-942,946-982 |
| LDLa | 946-987,1022-1060,1062-1099,1101-1038,1150-1189,1190-1231,1243-1280 |
| EGF | 1281-1314,1315-1353 |
| LY | 1411-1455,1505-1547 |
| EGF_CA | 1615-1651 |
| Transmembrane region | 1679-1698 |
| Cytoplasmic domain | 1699-1807 |
| YWTD domain | 310-544,618-851,1385-1584 |
| NPLF motif | 1748-1751 |
| LL motif | 1756-1757 |
| N-glycosylation sites | 79, 222, 229, 235, 421, 915, 1161, 1173, 1180, 1243,1284,1462,1575 |
