# Supplementary material for: Characterization of Vitellogenin and Vitellogenin Receptor of Conopomorpha sinensis Bradley and Their Responses to Sublethal Concentrations of Insecticide
Source: Front Physiol. 2018 Sep 11;9:1250. doi: 10.3389/fphys.2018.01250 (PMC6154279; doi:10.3389/fphys.2018.01250)

**Supplemental file 1.** Protein sequence of *Conopomorpha sinensis* vitellogenin with a color demarcation of important domains and motifs.

The N-terminal signal peptide is **bold** and underlined. Vitellogenin ([PS51211](https://prosite.expasy.org/doc/PS51211)) and VWFD ([PS51233](http://prosite.expasy.org/cgi-bin/prosite/nicedoc.pl?PS51233)) are highlighted in gray and yellow, respectively. The putative **R/KXXR/K** consensus subtilisin-like endoproteases cleavage sites, conserved GL/ICG motif and five conservative cysteine- residues at the carboxy-terminus are highlighted by red and purple and **bolded** fonts, respectively.
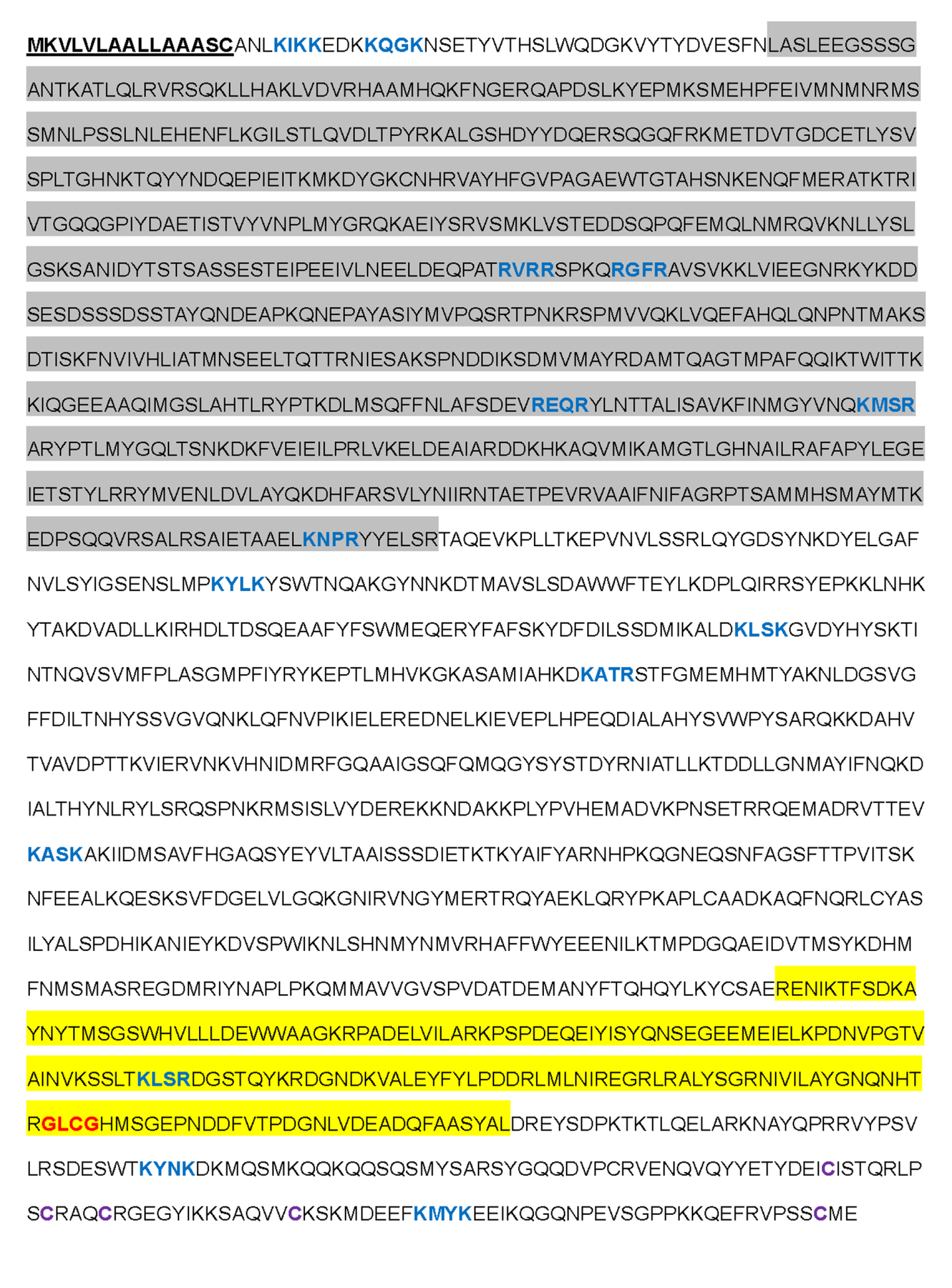

Supplement: Supplementary file 4 [file Data_Sheet_1.docx]
