# Supplementary material for: Characterization of Vitellogenin and Vitellogenin Receptor of Conopomorpha sinensis Bradley and Their Responses to Sublethal Concentrations of Insecticide
Source: Front Physiol. 2018 Sep 11;9:1250. doi: 10.3389/fphys.2018.01250 (PMC6154279; doi:10.3389/fphys.2018.01250)
Supplement: Supplementary file 5 [file Data_Sheet_2.docx]

**Supplementary file 2.** Nucleotide and protein sequence of vitellogenin receptor of *Conopomorpha sinensis*.

1. ATGTCTAACAAATGGCTGGTCACAATGATAACGGTGTCGCTGTGTGGTGTCGCATGGGCG

M S N K W L V T M I T V S L C G V A W A

61 GACTTCTCAGACGAGTACCAAACTTCGGACGACAGTTGTGTAGGCGAGGACGTGTTCCAG

D F S D E Y Q T S D D S C V G E D V F Q

121 TGCCACGACCGGGGCTGCGTGTCTCTGGACCAGTATTGCGACGGTCGTGACGATTGCGCC

C H D R G C V S L D Q Y C D G R D D C A

181 GATCGTTCGGACGAGAATTACTGTCCAAACCACCACGCTAAAATAGCAGCGTGCAACGCA

D R S D E N Y C P N H H A K I A A C N A

241 AGCCACGAGTACCTGTGCAAAGATGGCTCCAAGTGCATCCCTTACCCGTGGATGTGCAAC

S H E Y L C K D G S K C I P Y P W M C N

301 AACGAGCCGGAGTGTAGTGATGGAAGCGACGAGGCCGACTGCTCCTCCGTCGCTGCACCT

N E P E C S D G S D E A D C S S V A A P

361 AAGGAAGATGACCCGTGTCAAGGTTTCCAGTGCAGGAACGGTCAGTGCATATCCCGGCTG

K E D D P C Q G F Q C R N G Q C I S R L

421 TGGGTGTGCGACGGCGTCGTGGACTGTTACGACAGCAGTGATGAGTACAACGATGTGCTG

W V C D G V V D C Y D S S D E Y N D V L

481 TGCCGAACGCAGAAAGACTTCGAGCGCTACAGCGAGGTGGGCAGCATGACGTGCGAGCCG

C R T Q K D F E R Y S E V G S M T C E P

541 CAGGACTTCCTCTCCCACCGGCAGTACATGTGCGACGACCACTCCTTCTGTCTGCTGGAG

Q D F L S H R Q Y M C D D H S F C L L E

601 TACAGGATGTGTGACGGGGTCGCCGACTGCCGGGATCGAGGCGACGAGGGACCGTTTTGT

Y R M C D G V A D C R D R G D E G P F C

661 GCTAATTGGACCACAATGTGCAACAACGTAACATGCGGCCTGAACGCAACGTGCCGCCCC

A N W T T M C N N V T C G L N A T C R P

721 AACGCGTACGGGCCCACGTGCGAGTGCGACGTGAAGTACACGTACGACGTGCACACCGGC

N A Y G P T C E C D V K Y T Y D V H T G

781 GCGTGTGTGGACCGCGACGAGTGTGCCGCTGTCATGGCGGTGTGCGCGCAGAGGTGCGAC

A C V D R D E C A A V M A V C A Q R C D

841 AATTACAATGGCGGCTACAGTTGTACCTGCGATCCAGGGTATCGGAATGATTCGTTCATG

N Y N G G Y S C T C D P G Y R N D S F M

901 TGCTTTACTGCTAGTAAAGCGATAGAACCGCTCTTGATATTCAGCACTCGGAGGGACATT

C F T A S K A I E P L L I F S T R R D I

961 CGCTACATCAATCTGAAGACTAAGGCTATGGTCGTTGTAGCTTCTGATCTCAAACAGGCA

R Y I N L K T K A M V V V A S D L K Q A

1021 CATGGTGTGACCTACGACGGTTCATACTTGTATTGGGTGGAGACGGAGGCTGGACATCAG

H G V T Y D G S Y L Y W V E T E A G H Q

1081 GCAATTGTCCGGGCACAGCTGGACAACGTGCGGGGCACGAAGCAGACTTTAGTAGCGTTA

A I V R A Q L D N V R G T K Q T L V A L

1141 GGTCTAGAGCAACCAGGGGACATATCCGTGGACTGGCTCGCCGGCCATTTTTATTTCACG

G L E Q P G D I S V D W L A G H F Y F T

1201 GACTCGGCTCGTCGACACATCGCCGCGTGTCTACTAGATGGCTCTATATGTACCGTTCTC

D S A R R H I A A C L L D G S I C T V L

1261 AACACTACGGTGCATCATCCGAGGTTCTTAACACTACATCCGCAGGCTGGAGAAATGTAC

N T T V H H P R F L T L H P Q A G E M Y

1321 TGGTCAGACCACGACACCAATTCTGTGATAATGAAAGCCAACATGGACGGCTCCGGCGCC

W S D H D T N S V I M K A N M D G S G A

1381 CGCGTGTTCGTGGACAAACTGAGCAGCTTCGCCACCGGCCTCACTATTGACATTCCGGGC

R V F V D K L S S F A T G L T I D I P G

1441 ATGCGACTGTACTTTGTCGACAGAGGCTTGCATGTGGCGCCACTCGATGGACGCGGCCGT

M R L Y F V D R G L H V A P L D G R G R

1501 TATGTACTCCTGTCAGGGAATCTTCACCATCCTTACTCCGTGTCCGTGTACGAGACGGCG

Y V L L S G N L H H P Y S V S V Y E T A

1561 GCCTTCTTCAGCGACTGGGCGTCCAACAGCATCCAGCTCATCAACAAGATCATGCCCTCT

A F F S D W A S N S I Q L I N K I M P S

1621 GTCAGACGCAAGAGGATCGTGTCGGGGCTCGACATGCCGGTTTTAGGCATCCACATGTAC

V R R K R I V S G L D M P V L G I H M Y

1681 CACCCGATACTGATGCAGAAGTCACGCAGCGGCTGCGACGGGCACACCTGCTCGGAGCTG

H P I L M Q K S R S G C D G H T C S E L

1741 TGTTTGCCGCGCGGCGCGGGGTACGTGTGCGCGTGCTCGCAGGGACGCAAGCTTGTCAGC

C L P R G A G Y V C A C S Q G R K L V S

1801 AGGACTATGTGCTCTCCGGCGAGTTTCGCCGAGCTACCTCAGTTCCTGATCGTCGGTGGC

R T M C S P A S F A E L P Q F L I V G G

1861 GGTTCGCACTTCACTCGAGTGAGATACAACTCGCTGGGCAACCCGGAGAGCAGGGCCGTG

G S H F T R V R Y N S L G N P E S R A V

1921 AGGTTTGACATAGGACGAGTGCAGGCATTGGCTTACAATAACTTTAGACGTGTTCTGTAC

R F D I G R V Q A L A Y N N F R R V L Y

1981 GTGTACGACAGCCAAAGAAAATCAATAAATTTCATTCACATGAACAACTTCTCCACTGGA

V Y D S Q R K S I N F I H M N N F S T G

2041 GTGACGAATCTGCTCGCGTTCAAGCATTTGGAGAACGTCGTCGACATGGACTACGATTAC

V T N L L A F K H L E N V V D M D Y D Y

2101 GTAACAGACAACCTGTACGTCCTGGACTCCGGCCGCCAGACGCTGGAAGTGGTGAACACC

V T D N L Y V L D S G R Q T L E V V N T

2161 AAGTCGAAGGAGCGCGCAATAGTGCACAAGTTCAGAGAATACGTGCCAATTGCTCTCTCT

K S K E R A I V H K F R E Y V P I A L S

2221 GTTATGCCCGAGTATGGTCGAGCGATGGTGGCGCTGAAGAGCGAGGGTCCCGAAGGTGGG

V M P E Y G R A M V A L K S E G P E G G

2281 ATCCAGGTCGACTCCATAGGACTAAATGGTCACGAGAGAATTAATGTGGTCGAAAATTAT

I Q V D S I G L N G H E R I N V V E N Y

2341 TTACAAGGTCCGCAAGTGCGTCTCCGCTACTCCGGCCATGAGGATAAAGTGTACATATCG

L Q G P Q V R L R Y S G H E D K V Y I S

2401 GACGAAGGCAACGGTCGTATATATTCTATTCATCCATCAGGCACTGGCAAGGAGTTGTAC

D E G N G R I Y S I H P S G T G K E L Y

2461 CGTGACGTCTCCACCAAGATAACGAGCGTGGCGGTGTCCGACGACACGATATTCTGGACC

R D V S T K I T S V A V S D D T I F W T

2521 GACAGACACACGCCCCGACTGTTCTGGTCGCACGTCCACGACGTGTCCACAAACGTGCGA

D R H T P R L F W S H V H D V S T N V R

2581 CGGTTGGAGATGACAATATTCCCGAAGCACAGCCAGCTAATAATACAATCGACCTCTTTC

R L E M T I F P K H S Q L I I Q S T S F

2641 TTTCAAAGCTTAAAATCTCCCATCCTAACCCATCCCTGTTTCCAAACTAACCCGTGTTCG

F Q S L K S P I L T H P C F Q T N P C S

2701 CACGTGTGCACCCAAACCCCGCACCCAAAGCTGCCAAACACCAACAACACCTCAACCCCA

H V C T Q T P H P K L P N T N N T S T P

2761 ATCCCTAAAATGGGTTACATGTGCCTGTGTCCTCCGGGGATGGTGCTCATGGGCAACAAG

I P K M G Y M C L C P P G M V L M G N K

2821 TGCCAAGAGCTGAGCTCCTGTAAGAAGGATGAAGTGATATGCTTGAATGACAACAAATGT

C Q E L S S C K K D E V I C L N D N K C

2881 GTGGATGGACATATTTGTGATGGCGTCAAAGATTGTAAAGATGGCAGTGACGAGAATGGA

V D G H I C D G V K D C K D G S D E N G

2941 TGTGATCCGCAGTCTCAAGTGCCACCTCGCTGCGGACCCGAGCAGAAATTATGCCACAAT

C D P Q S Q V P P R C G P E Q K L C H N

3001 TTCTGCGTGGACAACAAAGTCGTTTGTGTCAGTAGTGATATACAACCTAACAATACTAAA

F C V D N K V V C V S S D I Q P N N T K

3061 TTGACATGTACTTCGGAGGAGTTCCAATGCCGCGCCAAGTGCATCCCGCGGTACAAGGTG

L T C T S E E F Q C R A K C I P R Y K V

3121 TGCGACCTCAGCGAAGACTGTCCTGGAGGAGAAGACGAGAGCGCCTTCTTGTGCCGGAAC

C D L S E D C P G G E D E S A F L C R N

3181 AATCTCTGTAGAGACGAAGAGTGGAGGTGTCAATCTGGAGCCTGCATACCATCGAGCTGG

N L C R D E E W R C Q S G A C I P S S W

3241 CGCTGCGACAGACACGGGGACTGCGCTGATGAGACTGATGAAGTTAACTGCGAATACGAA

R C D R H G D C A D E T D E V N C E Y E

3301 AAGTGCAAGGACGACGAGTGGCAGTGTGGCGACGGCTCGTGCATAGACTTCTCTCGTCGC

K C K D D E W Q C G D G S C I D F S R R

3361 TGCGACGACGTGCTCGACTGCGACGACCATTCCGACGAGGAGGCCTGCGACAACGACGAC

C D D V L D C D D H S D E E A C D N D D

3421 CTGGACGATTCACATTCAGAGGAGTCTCCTTGCGAAGATTTCGAGTACACATGCGCCATG

L D D S H S E E S P C E D F E Y T C A M

3481 AACAGAAGTATCTGCCTGCCACTAACTGCTGTATGTAACGGCACTTCGGAATGCCCGAAC

N R S I C L P L T A V C N G T S E C P N

3541 GGAACAGACGAAGCCGGCTGTGACAGCCTCTGCCCGCCCCACATGTTTCACTGCCGGGAT

G T D E A G C D S L C P P H M F H C R D

3601 GACCGGCTCTGCCTGCCCCCGAGGAAGGTCTGCGACGGGAAGGTGGACTGCAAAGATGGG

D R L C L P P R K V C D G K V D C K D G

3661 AGTGACGAGGCACCTAAAAATAGCTGCAATGCAACGCGTCCACCAACGCCGCCATATTTG

S D E A P K N S C N A T R P P T P P Y L

3721 ATGTCAAATTGTACCGGGAGGTACAGTTGCGGCTCAGGCGAGTGTGTCGACTTGCACGTC

M S N C T G R Y S C G S G E C V D L H V

3781 GTGTGCGACAGTAAAGCTGACTGCGCCGATGGTTCTGATGAGGGCGGGAAGTGTGACGAG

V C D S K A D C A D G S D E G G K C D E

3841 TCTTGCACGAACACATCGTGCTCGCTGGCGTGCCGCGCGACGCCTCGCGGCGAGCAGTGC

S C T N T S C S L A C R A T P R G E Q C

3901 GCGTGTCGCGCCGGGGCCGCGGCGCGCGCGGGCCGCTGTGTCGACATCAACGAGTGTGCT

A C R A G A A A R A G R C V D I N E C A

3961 TCATGGCCCTGTGCGCAGCGGTGTACCAATACTGAGGGGTCGTTTGTGTGTGGATGCTTT

S W P C A Q R C T N T E G S F V C G C F

4021 TGGGGGTATCGGTTGGGAACCGACGGTCGCAGATGCAAAGCGCTGTTCCCACCCAAAGTA

W G Y R L G T D G R R C K A L F P P K V

4081 ATAACGGCTACACCGGACGGAGAAATGAGTGATTCTACGGAAAAGTACTATTCCCGTGCG

I T A T P D G E M S D S T E K Y Y S R A

4141 CTGAAGCATATTTCCCTTGATTATAACCGAAGTTTGCTGTATGGGGTAACCCAGCAGGAT

L K H I S L D Y N R S L L Y G V T Q Q D

4201 GAGCTGGTGATGGCTGCCAGGGGTGGTACCAGGTCCTCCACTCTCATCAATCTTGGAACA

E L V M A A R G G T R S S T L I N L G T

4261 CCCACTGCGCTGGTCGTAGAATGGGTGACCGGAAACTTGTACTTCGCAACTCGAGTGGAC

P T A L V V E W V T G N L Y F A T R V D

4321 TCATACGCTAGACTCAACGTTTGTCATTTTGTATTGGAGAAGTGTGCCAGACTCGGTAAA

S Y A R L N V C H F V L E K C A R L G K

4381 CTTAACTCGACCCTGCCGCTGAACTCCGAAGTAACAACAATGGCAGTGGACCCATCAAGC

L N S T L P L N S E V T T M A V D P S S

4441 CACCGGCTATTCTACAGCGTGTACAAGGAAAAAGCGTCCGTATTGTACTGGTCTAGTCTA

H R L F Y S V Y K E K A S V L Y W S S L

4501 GTTGGCGAGAGGCCGCTGGTGCTCGCTGAACTGAAAGCGAATTGCACGGGATTGGCTGTC

V G E R P L V L A E L K A N C T G L A V

4561 GATATCAATAAAAGGAGAATTTATGTCGCACAAATGGGCGCCAGCATTATATTTCAGATT

D I N K R R I Y V A Q M G A S I I F Q I

4621 GGTTATGAAGGGAATGTAATGGCAATATTAACGCATCAGACATTCCTCAAGCGACCTCAC

G Y E G N V M A I L T H Q T F L K R P H

4681 ACCTTAACGCTATTGGCTGACGACATATATTTCCTTGAGCACAACACGAGTCAAATCAAC

T L T L L A D D I Y F L E H N T S Q I N

4741 TACTGCACATTTTTCGACGAGACATGCCACCCGTACGTACATCGCACTATGAATACGGAC

Y C T F F D E T C H P Y V H R T M N T D

4801 ACGTACGCACTGAGTCACCCTAGTATACAGAGGGGTGACTTGGTGAACGACTGTGCGGGC

T Y A L S H P S I Q R G D L V N D C A G

4861 AATAAGTGTGACAACTTGTGCGTGCCGAGCGATAATGGACCTAAATGTCTATGTTACGAT

N K C D N L C V P S D N G P K C L C Y D

4921 GGAAGTTTCGTTGAAAATGGCAAGGAGTGCACACTTGAAGGTTTGAGTGAGGTACCAAAG

G S F V E N G K E C T L E G L S E V P K

4981 TTCAAGTTCGTGTCTCTGCCTACCCCGTGGCGCTGGCGCTCCTCCCGCTCCTTCACAGTG

F K F V S L P T P W R W R S S R S F T V

5041 CCCGTGGTCGTGGTGCTGAGCATCGTCACCGCCCTCGGACTGTTCATCTTCCTGAGGAAG

P V V V V L S I V T A L G L F I F L R K

5101 AAGTGTAAACATGCTTTTACTACTGCAGTGCGTTTCCGAAACACATCGGCGTCCACTTCC

K C K H A F T T A V R F R N T S A S T S

5161 GGCGCAGACGACGCTGAAGCCACCGTGCACTTCCACACTGAGGACGGAGGCGTGCGACGT

G A D D A E A T V H F H T E D G G V R R

5221 CGTCGCTACGCTGAATACGTGAACCCGCTGCAGAACGTGCGCTCTCTGCTCGTAAACACT

R R Y A E Y V N P L Q N V R S L L V N T

5281 CTCTTCAGGAATAAACGACCTGTGGGAACAGCCGGTCTTCACATCGACGTGCCAACTCGA

L F R N K R P V G T A G L H I D V P T R

5341 GATGACCGCAGCACCACACCAACCAGCACAGCGTCTTCAGAACCAGACTACAAGGATACA

D D R S T T P T S T A S S E P D Y K D T

5401 AGGCAATTTCTTCCGAAGCCATAG

R Q F L P K P *
